# Sex Disparity in Patients with Gastric Cancer: A Systematic Review and Meta-Analysis

**DOI:** 10.1155/2022/1269435

**Published:** 2022-11-02

**Authors:** Xiaoyi Luan, Penghui Niu, Wanqing Wang, Lulu Zhao, Xiaojie Zhang, Dongbing Zhao, Yingtai Chen

**Affiliations:** National Cancer Center/National Clinical Research Center for Cancer/Cancer Hospital, Chinese Academy of Medical Sciences and Peking Union Medical College, 17 Panjiayuan Nanli, Beijing 100021, China

## Abstract

**Objective:**

This systematic review and meta-analysis aimed to ascertain whether sex-based differences influence clinicopathological characteristics and survival outcomes of gastric cancer patients.

**Background:**

Gastric cancer in females has received less attention than in males. Clinicopathological features and survival outcomes of females with gastric cancer have been reported in several studies with controversial results.

**Methods:**

We systematically reviewed clinical studies from PubMed, Cochrane Library, Embase, and Web of Science published up to June 2022. The effect sizes of the included studies were estimated using odds ratios (ORs). Heterogeneity was investigated using the *χ*2 and *I*^2^ tests, while sensitivity analyses were performed to identify the source of substantial heterogeneity. All data used in this study were obtained from previously published studies obviating the need for ethical approval and patient consent.

**Results:**

Seventy-six studies with 775,003 gastric cancer patients were included in the meta-analysis. Gastric cancer patients were less likely to be females (*P* < 0.00001). Female patients were younger in age (*P* < 0.00001) and showed a higher percentage of distal (*P* < 0.00001), non-cardia (*P* < 0.00001), undifferentiated (*P* < 0.00001), diffuse (*P* < 0.00001), and signet-ring cell carcinoma (*P* < 0.00001). Female patients showed better prognosis in both 3-year (*P* = 0.0003) and 5-year overall survival (OS) (*P* < 0.00001), especially White patients. However, females were associated with lower 5-year OS relative to males in the younger patients (*P* = 0.0001).

**Conclusions:**

In conclusion, gender differences were observed in clinicopathological characteristics and survival outcomes of gastric cancer. Different management of therapy will become necessary for different genders.

## 1. Introduction

Gastric cancer is the fifth most common cancer globally and the fourth leading cause of cancer‐related mortality [1]. Gastric cancer is more common in males than females [2]. Many studies have concluded that exposure to estrogen reduces the risk of gastric cancer [3–6]. Some studies showed sex disparity may play a special role in the development of cardia and intestinal type of gastric cancer [7, 8]. As research on sex-related differences in gastric cancers has progressed, there has also been a concomitant interest in female gastric cancer research.

Studies on the prognosis of gastric cancer in females have produced mixed results. While in most studies, female patients had a better prognosis [9–18], several other studies showed no independent sex-related associations with OS [19–22]. Though some recent studies have found that females had a better overall prognosis, this was not found to be the case in young female patients [23–25].

As such, the aim of the current study was to compare the clinicopathological characteristics and survival outcomes of female and male patients with gastric cancer through systematic review and meta-analysis, thus providing evidence suggesting the need for specific treatments optimized for female and male gastric cancer patients.

## 2. Methods

### 2.1. Search Strategy

Two investigators independently and systematically searched PubMed, Embase, Cochrane Library, and Web of Science databases for clinical studies using the following search terms: “gastric” or “stomach,” “cancer” or “neoplasm,” “women” or “females” or “girls,” “sex” or “gender.” All articles published in English were included since the establishment of the database until the end of June 2022. Reference lists of the relevant systematic reviews and meta-analyses were also screened for other potential articles that might have been missed in the database search.

### 2.2. Inclusion and Exclusion Criteria

The eligibility criteria for inclusion were as follows: (I) studies compared females and male patients with gastric cancer; (II) studies contained quantitative clinicopathological characteristic information; and (III) studies involved at least one of the survival outcomes mentioned.

The exclusion criteria were as follows: (I) abstract form only, letters, editorials, expert opinions, case reports, and studies lacking control groups; (II) duplicate research based on author or center; (III) data were inappropriate or unextractable; (IV) studies of benign lesions and special types of gastric cancer; (V) patients in the study had other diseases or cancers that affected their hormone levels; and (VI) studies involved other strong confounding factors.

### 2.3. Data Extraction

All data from the included studies were independently extracted by two investigators. We extracted data on studies' authors, year of publication, study sites, document type, sample size, date sources, design, and quality assessment. The clinicopathological characteristics extracted from patients included sex, age, tumor size, tumor location, differentiation, histologic grading, Lauren type, Borrmann classification, the state of lymph node metastasis, pathologic tumor-node-metastasis (pTNM) stage, history of *Helicobacter pylori* (HP) infection, and family history. The survival outcomes included short or long-term survival rates on total population, different ethnic group, and different age group. Some data were extracted by Engauge Digitizer version 11.3 from the graphical survival plots when data were only available as Kaplan–Meier curves. The stage of gastric cancer in the systematic review and meta-analysis was performed according to the American Joint Committee on Cancer (AJCC) tumor-node-metastasis (TNM) staging system. Discrepancies in data extraction were resolved through discussion by the two investigators.

### 2.4. Quality Assessment

Two investigators used the Newcastle-Ottawa Quality Assessment Scale (NOS) to evaluate the methodological quality of the included studies [26]. The NOS scores range from 0 to 9 and studies with NOS score ≥6 are considered high-quality studies. Discrepancies in quality assessment were resolved through discussion by the two investigators.

### 2.5. Statistical Analysis

We assessed heterogeneity between studies using both the *I*^2^ test and the *χ*^2^ test. The *I*^2^ test and *χ*^2^ test were the methods to test for heterogeneity in multiple independent studies and were often used in meta-analysis. Heterogeneity was considered significant when *I*^2^ values over 50% and the *χ*^2^ test with a *P* value < 0.10 [27] were obtained. Review Manager V.5.4.1 (Cochrane Collaboration, Oxford, United Kingdom) was used to conduct the systematic review and meta-analysis by generating forest plots. We set confidence intervals (CIs) at 95%. Results were expressed as odds ratios (ORs) with corresponding 95% CI by using the Mantel–Haenszel method for dichotomous outcomes and weighted mean difference (WMD) with corresponding 95% CI for continuous variables. Hazard ratio (HR) with corresponding 95% CI was used to assess the survival outcomes. The random effects model was used when significant heterogeneity obviously existed; otherwise, the fixed effects model was used [28, 29]. It was necessary to identify sources of significant heterogeneity by sensitivity analysis.

## 3. Results

### 3.1. Study Selection

**Figure 1:**
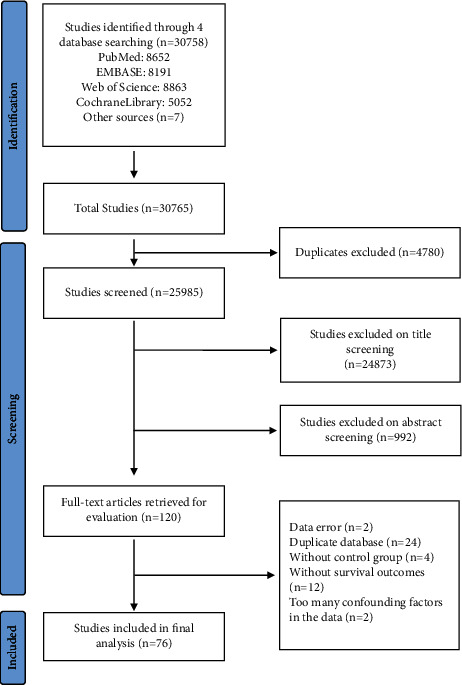
The flowchart of the research process until June 2022.

Figure 1 shows the flow sheet of the search process. A total of 30,765 relevant clinical studies were identified with our search strategy. After initial screening of titles and abstracts, 120 potentially eligible articles were retrieved by a full-text review. Articles were then based on exclusion and inclusion criteria. Finally, 76 studies with 775,003 gastric cancer patients were included in the systematic review and meta-analysis for further investigation, of which two were prospective studies, twenty-nine were observational studies, and the rest were retrospective comparative studies.  Table 1 shows the essential characteristics and the NOS scores of the included studies.  Table S1 shows the clinicopathological characteristics of the included studies.

### 3.2. Clinicopathological Characteristics

Clinicopathological characteristics of the gastric cancer patients are presented in Table 2 and S1. Gastric cancer patients were less likely to be females (OR = 0.27, 95% CI: 0.26, 0.29, *P* < 0.00001, *I*^2^ = 99%) (Figure S1). Compared with the male patients, female patients were younger in age (WMD = −2.57, 95% CI: −3.06, −2.09, *P* < 0.00001, *I*^2^ = 45%) and showed a higher percentage of distal (OR = 1.41, 95% CI: 1.24, 1.60, *P* < 0.00001, *I*^2^ = 96%), overlapping (OR = 1.64, 95% CI: 1.02, 2.63, *P* = 0.04, *I*^2^ = 98%), non-cardia (OR = 1.46, 95% CI: 1.26, 1.70, *P* < 0.00001, *I*^2^ = 99%), undifferentiated (OR = 2.3, 95% CI: 1.98, 2.68, *P* < 0.00001, *I*^2^ = 67%), diffuse (OR = 1.87, 95% CI: 1.70, 2.06, *P* < 0.00001, *I*^2^ = 90%), and signet-ring cell carcinoma (OR = 1.76, 95% CI: 1.55, 1.99, *P* < 0.00001, *I*^2^ = 84%) (Figures S2 and S3). Female patients were more likely to have a history of HP infection (OR = 1.16, 95% CI: 1.03, 1.31, *P* = 0.02, *I*^2^ = 21%) (Figure S4).

### 3.3. Postoperative Complications

A total of 2,912 patients from three studies exhibited postoperative complications [53, 66, 84]. The meta-analysis revealed that the complication rate was lower in female patients than in male patients (OR = 0.78, 95% CI: 0.66, 0.93, *P* = 0.005, *I*^2^ = 0%) (Figure S5).

### 3.4. Survival Outcomes

**Figure 2:**
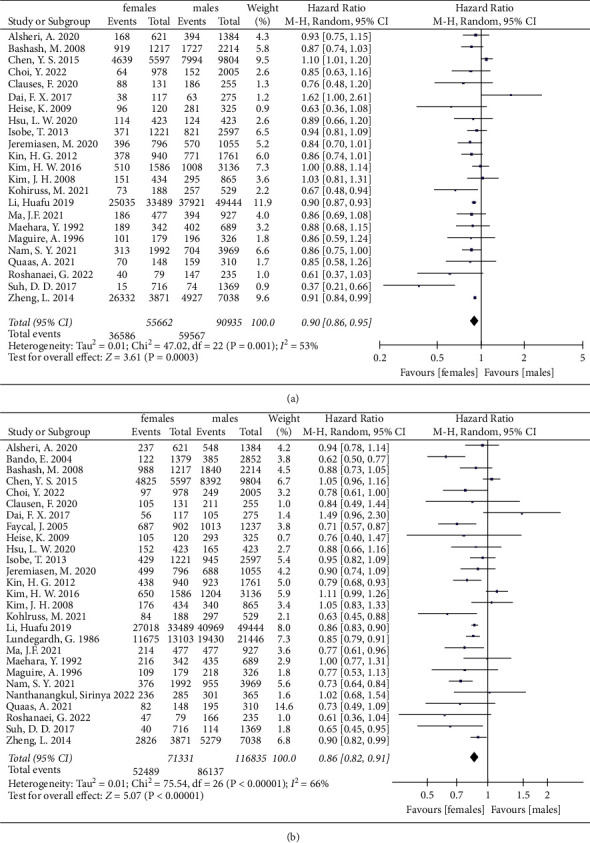
The 3-year and 5-year overall survival for gastric cancer between female and male groups. (a) The 3-year overall survival of total patients. (b) The 5-year overall survival of total patients.

Survival outcomes of the gastric cancer patients are presented in Table 3.  Figure 2 presents the meta-analysis of the 3-year overall survival (OS) and 5-year OS in the total patient population reviewed. Significant sex-based differences in the OS of the total patient population obtained from the twenty-eight studies reviewed were found [9–16, 19–24, 31, 45, 55, 61, 65, 69–72, 80, 81, 86, 87, 89]. Our meta-analysis showed that females with gastric cancer were associated with better 3-year OS and 5-year OS relative to males (HR = 0.90, 95% CI: 0.86, 0.95, *P* = 0.003, *I*^2^ = 53%; HR = 0.86, 95% CI: 0.82, 0.91, *P* < 0.00001, *I*^2^ = 66%,respectively).

**Figure 3:**
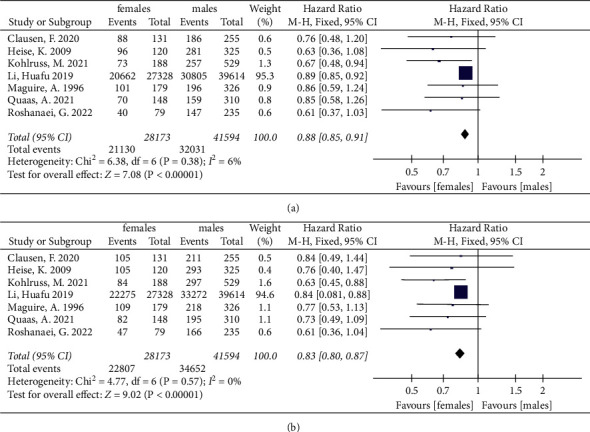
The 3-year and 5-year overall survival for gastric cancer between female and male groups among White gastric cancer patients. (a) The 3-year overall survival of White patients. (b) The 5-year overall survival of White patients.

In addition, further survival analyses between female and male patients were done with different ethnic groups. Among White gastric cancer patients, females showed a better prognosis compared with males (HR = 0.88, 95% CI: 0.85, 0.91, *P* < 0.00001, *I*^2^ = 6%; HR = 0.83, 95% CI: 0.80, 0.87, *P* < 0.00001, *I*^2^ = 0%,respectively) (Figure 3). However, no significant differences between the female and male groups' OS in Asian gastric cancer patients were found (HR = 0.95, 95% CI: 0.88, 1.03, *P* = 0.26, *I*^2^ = 65%; HR = 0.92, 95% CI: 0.84, 1.01, *P* = 0.08, *I*^2^ = 77%) (Figure S6).

**Figure 4:**
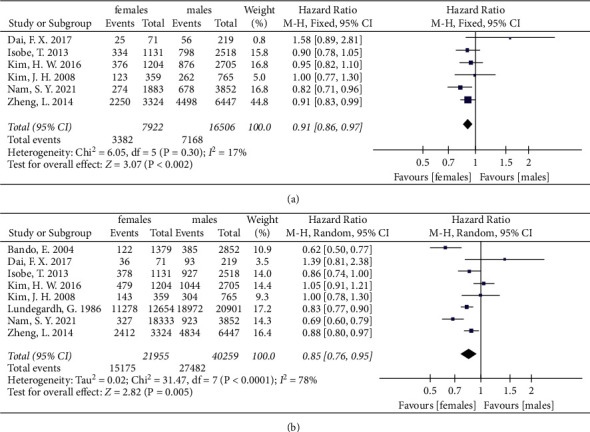
The 3-year and 5-year overall survival for gastric cancer between female and male groups in older patients. (a) The 3-year overall survival in older patients. (b) The 5-year overall survival in older patients.

**Figure 5:**
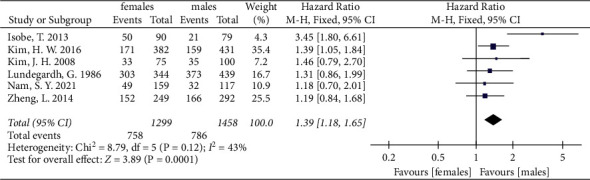
The 5-year overall survival for gastric cancer between female and male groups in younger patients.

We also divided female and male patients into two groups by age. Due to the limitation of meta-analysis, different articles have different age criteria. So, the age group was blurred in this paper. The patients were divided into two groups with 40–50 years old as the dividing line based on previously published studies and data. Most articles used 40 or 45 years old as the dividing line [24, 31, 55, 61, 69, 86]. One article used 50 years old as the cutoff [71]. Only patient data from those older than 55 years were used from the article by Bando et al. [45]. In older patients, the pooled 6 and 8 studies, respectively, showed that females had a better prognosis in both 3-year and 5-year OS (HR = 0.91, 95% CI: 0.86, 0.97, *P* = 0.002, *I*^2^ = 17%; HR = 0.85, 95% CI: 0.76, 0.95, *P* = 0.005, *I*^2^ = 78%) (Figure 4). In contrast, females were associated with lower 5-year OS relative to males in young patients (HR = 1.39, 95% CI: 1.18, 1.65, *P* = 0.0001, *I*^2^ = 43%) (Figure 5).

### 3.5. Metastasis

Nine of the seventy-six studies reported the metastasis of gastric cancer [7, 13, 15, 22, 23, 37, 69, 79, 85]. The result showed that females with gastric cancer were less likely to develop hepatic metastasis than males (OR = 0.56, 95% CI: 0.47, 0.67, *P* < 0.00001, *I*^2^ = 0%). Sex-related differences were not found in lymphovascular invasion, lymph node metastasis, or perineural metastasis (*P* > 0.05) (Figure S7).

## 4. Discussion

This meta-analysis was conducted utilizing data from 43 retrospective comparative trails [7, 9, 12–16, 20, 24, 30, 32–39, 41, 42, 45, 48–51, 53, 55, 64, 66, 69–72, 74, 79–81, 84–89], two prospective studies [44, 83], and thirty-one observational studies [10, 11, 19, 21–23, 25, 31, 40, 43, 46, 47, 52, 54, 56–63, 65, 67, 68, 73, 75–78, 82] with 775,003 gastric cancer patients. The results revealed that the prognosis of female gastric cancer patients was better than that of males for total patients, but there was no significant difference in the Asian patient group. The results were even reversed in younger patients. To the best of our knowledge, this meta-analysis is the first to evaluate differences in clinicopathological characteristics and prognosis between female and male patients.

Our study showed that the incidence of gastric cancer is lower in females than in males. While the exact physiological mechanism is unclear, it had been suggested that female hormones could reduce the risk of gastric cancer. The prevailing view of the past was that frequent exposure to environmental carcinogens might lead to a predominance of gastric cancer in males, such as cigarettes [90]. But as the research went on, differential exposure to established risk factors cannot totally explain the differences. Several studies revealed that the use of exogenous hormones also played a protective role in gastric cancer risk, which suggested a high correlation between gastric cancer and hormones [3–5]. Our study found that females with gastric cancer were younger in age compared with males. Other studies have also reported higher incidence of gastric cancer in younger females [24, 55, 91]. This trait was also believed to be related to hormonal factors. Higher estrogen levels and a higher proportion of estrogen receptor positive cells have been found in younger females [30, 35, 92]. Therefore, more studies are needed to explore the role of hormones in gastric cancer.

Some findings of clinicopathological features in the meta-analysis were consistent with previous studies, including a higher proportion of distal, non-cardia, undifferentiation, diffuse histology, and signet-ring cell carcinoma in female patients. Many studies suggested a possible suppressive role of female sex hormones on cardia cancer and intestinal gastric cancer [7, 8, 93]. However, it has recently been suggested that estrogen can promote the development of undifferentiated and diffuse gastric cancer. The estrogen receptor (ER) positive rate has been reported to be slightly higher in young females and in poorly differentiated gastric cancer. This may be the reason that poorly differentiated histological results have been found more common in female gastric cancer patients [30, 35, 94, 95]. One study detailed the tumorigenic mechanism of estrogen in the development of ER*α*-positive diffuse-type gastric adenocarcinoma [94]. In addition, HP infection seemed to be involved in this process. CagA + HP infection is associated with an increased risk of distal gastric cancer [96–98]. A study of 917 patients with gastric cancer reported a higher titer of HP antibody in diffuse gastric cancer than in intestinal type, suggesting that HP might be more closely related to diffuse gastric cancer [99]. Furthermore, one study showed that HP could secrete a type of toxin called CagA, which might enhance the effect of estrogen on diffuse gastric cancer [94]. This may explain why studies found no sex-related differences in HP infection; however, our study found that female gastric cancer patients were more likely to have a history of HP infection. Therefore, young females with physiologically high levels of estrogen showed a higher percentage of distal, non-cardia, undifferentiation, diffuse, and signet-ring cell carcinoma under the influence of HP.

For postoperative complications, our study showed that being male was a risk factor for an adverse outcome. One study found that men are more likely to get infections after surgery [100]. Some research found that postoperative complications may be related to differing female/male patterns of adipose tissue distribution [53]. Visceral obesity but not general obesity was significant independent factor associated with postoperative complications in males [66]. Furthermore, the higher postoperative complications in males might be due to higher preoperative complications and more extensive surgical procedures [84]. However, other studies showed that the level of sex hormones was related with postoperative complications rather than sex-related differences. In both sexes, higher levels of 17*β*-estradiol predicted a poor prognosis [101, 102]. Therefore, more studies are needed to research the relationship between sex and postoperative complications in the future. If differences do indeed exist, different management of therapy will become necessary for the different genders [101].

Female gastric cancer patients had a better prognosis, and this finding was consistent with most previous studies [9–18]. There are many possible reasons for this phenomenon. Firstly, females were more likely to have non-cardia and distal gastric cancer than males. Many studies have found that cardiac and proximal gastric cancer were more advanced and showed a poorer prognosis [103–105]. Secondly, our study included a large number of White gastric cancer patients, and White females have been shown to have a better prognosis in previous studies [17, 80]. Thirdly, one study found that the ATRX gene was found to mutate more frequently in female gastric cancer patients. The ATRX gene is a protein coding gene associated with alpha-thalassemia myelodysplasia syndrome and intellectual disability-hypotonic facies syndrome, X-linked. Female patients with ATRX mutation obtained significantly better survival benefits after treatment with immune checkpoint inhibitors [106]. Fourthly, survival was significantly increased in females receiving neoadjuvant chemotherapy, especially in females with microsatellite instability-high (MSI-H) tumors [15]. In terms of tumor metastasis, females were less likely to develop hepatic metastasis, consistent with previous studies [13]. One possible reason could be that estrogen has an anti-cancer effect in some non-target organs such as the liver and colon, but more research is needed to prove the exact mechanism [107].

Other studies have found that sex-related differences have different influences on the prognosis of gastric cancer patients in different racial groups, and this study reached the same conclusion. The reason for this phenomenon can be explained by the differences in molecular mechanisms between the two sexes among gastric cancer patients of different ethnic groups [80]. Moreover, female gastric cancer patients might have a worse prognosis under the influence of HP as previously mentioned. This phenomenon could also be explained by the higher prevalence of HP infection in under-developed and developing countries than in developed countries [108]. Note, however, that in one previous study, females showed a worse prognosis compared with males among Asian gastric cancer patients [80]. In our meta-analysis, there was no significant difference in survival outcomes between Asian males and females, which was consistent with another study [17]. Therefore, the effect of sex-related differences on the prognosis of Asian gastric cancer patients needs to be further studied.

In this study, younger female gastric cancer patients were found to have a lower 5-year OS relative to younger male patients. Many studies reached similar conclusions when age was analyzed [23, 24, 55, 69]. Poorly differentiated adenocarcinoma was more likely to be identified in younger women, which might be related to hormone levels [69, 84]. The poorly differentiated tumor types in females might partly explain the poor prognosis. Studies have also found that younger female patients had larger tumors and more advanced TNM staging and were also likely to develop lymph node metastases [72, 79]. Another study found that young female patients showed lower OS than male patients in both signet-ring cell and non-signet-ring cell histological types, which partly supports this view [69].

There were several limitations in our meta-analysis. First of all, most of the studies we included were retrospective studies with some limitations, which were at risk of publication bias and heterogeneity. Secondly, we were not able to analyze information from Black gastric cancer patients because most of the studies meeting inclusion criteria used in this study were only related to clinical characteristics and survival outcomes in White and Asian patients. Thirdly, the lack of available patient data did not allow our analysis to assess disease-specific survival and disease-free survival, and we could only deal vaguely with different versions of TNM stages. Furthermore, heterogeneities in the included studies' female/male ratios, tumor location, Lauren type, and other variables were all significant. Despite these limitations, this meta-analysis to our knowledge is the first to evaluate differences in clinicopathological characteristics and prognosis between female and male patients. Moreover, we assessed the effect of sex-related differences on postoperative complications and metastasis. In addition, all clinical studies included in this meta-analysis were of high quality, which may provide clinicians with more valuable resources for patient management and decision making.

## 5. Conclusions

In conclusion, gender differences were found in clinicopathological characteristics and survival outcomes of gastric cancer patients. Female patients with gastric cancer were more often diagnosed with distal, non-cardia, undifferentiated, and signet-ring cell carcinoma. The clinicopathological type of tumor in female patients with gastric cancer was more aggressive than that found in males. Female patients showed better prognosis especially in the White patients' group. However, the results were reversed in younger patients. We expect to see more studies researching the molecular mechanisms and pathophysiological process relationships between gastric cancer prognosis in female and male populations. We will further analyze sex-related differences among different regions and ethnic groups (including Black, White, and Asian) in our next study. Furthermore, we will distinguish sex-related differences among different age subgroups of gastric cancer in subsequent studies, so as to explore the impact of those differences in gastric cancer between young, middle-aged, and elderly groups on prognosis and explore the impact on women before and after menopause on prognosis. We also look forward to the publication of studies with larger sample sizes in the future to confirm our findings.

## Figures and Tables

**Table 1:** Basic characteristics of the included 76 studies.

Authors	Year	Country/region	Date sources	Document type	NOS	No.	Group		Age	Tumor location		TNM stage			
										Proximal	Distal	I	II	III	IV
Tokunaga et al. [30]	1986	Japan	—	Retrospective comparative study	6	86	F	34	—	—	—	—	—	—	—
							M	52	—	—	—	—	—	—	—
Lundegårdh et al. [31]	1986	Sweden	Community	Observational study	7	34548	F	13102	—	—	—	—	—	—	—
							M	21446	—	—	—	—	—	—	—
Sipponen et al. [32]	1988	Finland	Hospital	Retrospective comparative study	7	532	F	250	—	154	96	—	—	—	—
							M	282	—	154	128	—	—	—	—
Hirose et al. [33]	1989	Japan	Hospital	Retrospective comparative study	7	1242	F	454	—	—	—	—	—	—	—
							M	788	—	—	—	—	—	—	—
Janssen et al. [34]	1991	Norway	Hospital	Retrospective comparative study	8	375	F	141	—	—	—	—	—	—	—
							M	234	—	—	—	—	—	—	—
Matsui et al. [35]	1992	Japan	Hospital	Retrospective comparative study	6	107	F	42	—	—	—	—	—	—	—
							M	65	—	—	—	—	—	—	—
Maehara et al. [23]	1992	Japan	Hospital	Observational study	7	1031	F	342	55.5 ± 13.9	86	256	—	—	—	—
							M	689	58.9 ± 11.4	207	482	—	—	—	—
Furukawa et al. [36]	1994	Japan	Hospital	Retrospective comparative study	7	121	F	64	—	—	—	—	—	—	—
							M	57	—	—	—	—	—	—	—
Maeta et al. [37]	1995	Japan	Hospital	Retrospective comparative study	7	2325	F	856	—	—	—	—	—	—	—
							M	1469	—	—	—	—	—	—	—
Wu et al. [38]	1996	China, Taiwan	Hospital	Retrospective comparative study	6	536	F	94	—	—	—	—	—	—	—
							M	442	—	—	—	—	—	—	—
Maguire et al. [9]	1996	Spain	Hospital	Retrospective comparative study	8	851	F	304	—	—	—	—	—	—	—
							M	547	—	—	—	—	—	—	—
Galetsky et al. [39]	1997	Russia	Hospital	Retrospective comparative study	7	184	F	83	—	11	69	—	—	—	—
							M	101	—	37	61	—	—	—	—
Hansen et al. [40]	1997	Norway	Community	Observational study	7	38716	F	15485	—	1338	6020	—	—	—	—
							M	23231	—	3048	8241	—	—	—	—
Koriyama et al. [41]	2001	Brazil, Japan	Hospital	Retrospective comparative study	7	2314	F	824	—	74	750	—	—	—	—
							M	1490	—	204	1286	—	—	—	—
Corvalan et al. [42]	2001	Chile	Hospital	Retrospective comparative study	7	185	F	64	—	22	42	—	—	—	—
							M	121	—	45	73	—	—	—	—
Newnham et al. [25]	2003	England	Community	Observational study	6	21287	F	8378	—	—	—	—	—	—	—
							M	12909	—	—	—	—	—	—	—
Bani-Hani et al. [43]	2004	Jordan	Hospital	Observational study	7	201	F	73	—	—	—	—	—	—	—
							M	128	—	—	—	—	—	—	—
Tanaka et al. [44]	2004	Japan	Community	Prospective study	8	83	F	23	—	3	20	—	—	—	—
							M	60	—	11	49	—	—	—	—
Bando et al. [45]	2004	Japan	Hospital	Retrospective comparative study	7	4231	F	1379	—	—	—	—	—	—	—
							M	2852	—	—	—	—	—	—	—
Alipov et al. [46]	2005	Kazakhstan	Hospital	Observational study	6	139	F	53	—	—	—	—	—	—	—
							M	86	—	—	—	—	—	—	—
Herrera-Goepfert et al. [47]	2005	Mexico	Hospital	Observational study	7	330	F	157	—	13	143	—	—	—	—
							M	173	—	31	141	—	—	—	—
Faycal et al. [10]	2005	France	Community	Observational study	7	2139	F	902	—	—	—	—	—	—	—
							M	1237	—	—	—	—	—	—	—
Sasao et al. [48]	2006	Japan	Hospital	Retrospective comparative study	6	134	F	53	—	—	—	—	—	—	—
							M	81	—	—	—	—	—	—	—
Gwak et al. [49]	2007	South Korea	Hospital	Retrospective comparative study	6	621	F	212	56.5 ± 13.0	—	—	—	—	—	—
							M	409	57.9 ± 11.8	—	—	—	—	—	—
Bashash et al. [19]	2008	Canada	Community	Observational study	6	3431	F	1217	—	—	—	—	—	—	—
							M	2214	—	—	—	—	—	—	—
Kim et al. [24]	2008	South Korea	Hospital	Retrospective comparative study	7	1299	F	434	—	39	384	197	61	123	53
							M	865	—	100	754	321	104	230	210
Heise et al. [11]	2009	Chile	Community	Observational study	7	529	F	164	—	35	57	1	11	15	83
							M	365	—	123	122	8	9	38	214
Yu, and Zhao. [50]	2009	China	Hospital	Retrospective comparative study	7	351	F	103	—	—	—	—	—	—	—
							M	248	—	—	—	—	—	—	—
Sato et al. [51]	2009	Japan	Community	Retrospective comparative study	7	72789	F	25254	—	—	—	—	—	—	—
							M	47535	—	—	—	—	—	—	—
Mandong et al. [52]	2010	Nigeria	Hospital	Observational study	6	205	F	60	—	25	35	—	—	—	—
							M	145	—	45	100	—	—	—	—
Kim et al. [12]	2012	South Korea	Hospital	Retrospective comparative study	7	2701	F	940	—	72	857	444	194	255	47
							M	1761	—	181	1565	797	367	485	112
Lee et al. [53]	2012	South Korea	Hospital	Retrospective comparative study	8	243	F	107	—	—	—	80	27	—	—
							M	136	—	—	—	113	23	—	—
Coupland et al. [54]	2012	England	Community	Observational study	7	71929	F	25614	—	—	—	—	—	—	—
							M	46315	—	—	—	—	—	—	—
Isobe et al. [55]	2013	Japan	Hospital	Retrospective comparative study	7	3818	F	1221	—	—	—	—	—	—	—
							M	2597	—	—	—	—	—	—	—
Saha et al. [56]	2013	India	Community	Observational study	8	462	F	122	—	24	84	—	—	—	—
							M	340	—	52	242	—	—	—	—
Jiexian et al. [57]	2013	China	Hospital	Observational study	6	389	F	108	—	—	—	—	—	—	—
							M	281	—	—	—	—	—	—	—
Chen et al. [58]	2013	China, Taiwan	Community	Observational study	7	31524	F	10623	—	—	—	—	—	—	—
							M	20901	—	—	—	—	—	—	—
Liu et al. [59]	2013	China	Community	Observational study	7	4737	F	1563	—	—	—	—	—	—	—
							M	3174	—	—	—	—	—	—	—
Yan et al. [60]	2014	China	Hospital	Observational study	7	2379	F	511	—	—	—	—	—	—	—
							M	1868	—	—	—	—	—	—	—
Zheng et al. [61]	2014	China	Community	Observational study	7	10909	F	3871	—	418	1530	207	364	451	522
							M	7038	—	1124	2705	394	721	902	982
Dassen et al. [62]	2014	Netherlands	Community	Observational study	7	47295	F	17582	—	—	—	—	—	—	—
							M	29713	—	—	—	—	—	—	—
Feller et al. [63]	2015	Switzerland	Community	Observational study	6	15484	F	6236	—	—	—	—	—	—	—
							M	9248	—	—	—	—	—	—	—
da Costa et al. [64]	2015	Brazil	Hospital	Retrospective comparative study	6	127	F	44	—	—	—	—	—	—	—
							M	83	—	—	—	—	—	—	—
Chen et al. [65]	2015	China	Community	Observational study	6	15401	F	5597	—	—	—	—	—	—	—
							M	9804	—	—	—	—	—	—	—
Go et al. [66]	2015	South Korea	Hospital	Retrospective comparative study	7	597	F	219	—	—	—	182	26	11	—
							M	378	—	—	—	340	36	2	—
Jaehn et al. [67]	2016	Germany	Community	Observational study	6	4985	F	2260	—	—	—	—	—	—	—
							M	2725	—	—	—	—	—	—	—
Sierra et al. [68]	2016	Central and South America	Community	Observational study	6	27361	F	10869	—	—	—	—	—	—	—
							M	16492	—	—	—	—	—	—	—
Kim et al. [69]	2016	South Korea	Hospital	Retrospective comparative study	8	4722	F	1586	55.0 ± 13.0	—	—	897	65	623	—
							M	3136	57.9 ± 11.2	—	—	1858	119	1159	—
Nanthanangkul, Sirinya et al. [70]	2016	Thailand	Community	Retrospective comparative study	6	650	F	285	—	—	—	—	—	—	—
							M	365	—	—	—	—	—	—	—
Dai et al. [71]	2017	China	Hospital	Retrospective comparative study	6	392	F	117	—	—	—	—	—	—	—
							M	275	—	—	—	—	—	—	—
Suh et al. [72]	2017	South Korea	Hospital	Retrospective comparative study	7	2085	F	716	—	—	—	—	—	—	—
							M	1369	—	—	—	—	—	—	—
Liang et al. [73]	2017	China	Community	Observational study	6	5108	F	1331	—	787	390	—	—	—	—
							M	3777	—	2512	905	—	—	—	—
Jukic et al. [74]	2017	Croatia	—	Retrospective comparative study	6	60	F	26	69.8 ± 13.8	—	—	—	—	—	—
							M	34	69.3 ± 10.5	—	—	—	—	—	—
Bringeland et al. [75]	2017	Norway	Community	Observational study	6	878	F	323	—	—	—	—	—	—	—
							M	555	—	—	—	—	—	—	—
Kim et al. [7]	2018	South Korea	Hospital	Retrospective comparative study	7	758	F	227	57.1 ± 13.8	9	218	137	45	39	6
							M	531	57.7 ± 10.7	70	461	328	80	117	6
Anderson et al. [76]	2018	America	Community	Observational study	8	142783	F	63746	—	20553	31408	—	—	—	—
							M	79037	—	67920	36288	—	—	—	—
Lagergren et al. [77]	2018	Sweden	Community	Observational study	7	50263	F	18964	—	—	—	—	—	—	—
							M	31299	—	—	—	—	—	—	—
Jenabi et al. [78]	2019	Iran	Community, hospital	Observational study	6	5240	F	1420	—	—	—	—	—	—	—
							M	3820	—	—	—	—	—	—	—
Ryu et al. [79]	2019	South Korea	Hospital	Retrospective comparative study	8	1076	F	334	—	—	—	—	—	—	—
							M	742	—	—	—	—	—	—	—
Li et al. [80]	2019	China, America	Community, hospital	Retrospective comparative study	8	15991	F	6161	—	918	3857	449	1597	3907	211
							M	9830	—	2068	5259	639	2930	5913	348
Clausen et al. [81]	2020	Germany	Hospital	Retrospective comparative study	8	449	F	164	—	39	125	29	42	54	39
							M	285	—	104	172	45	59	135	46
Xiong et al. [82]	2020	China	Hospital	Observational study	7	19668	F	6195	—	850	1515	—	—	—	—
							M	13473	—	3516	2750	—	—	—	—
Alshehri et al. [20]	2020	South Korea	Hospital	Retrospective comparative study	7	2005	F	621	—	—	—	—	—	—	—
							M	1384	—	—	—	—	—	—	—
Hsu et al. [13]	2020	China Taiwan	Hospital	Retrospective comparative study	8	2673	F	694	—	—	—	—	—	—	—
							M	1979	—	—	—	—	—	—	—
Jeremiasen et al. [21]	2020	Sweden	Community	Observational study	6	1851	F	796	—	—	—	—	—	—	—
							M	1055	—	—	—	—	—	—	—
Atsumi et al. [83]	2021	Japan	Hospital	Prospective study	7	47	F	9	—	—	—	—	—	—	—
							M	38	—	—	—	—	—	—	—
Kalff et al. [84]	2021	Netherlands	Hospital	Retrospective comparative study	8	2072	F	768	—	—	—	—	—	—	—
							M	1304	—	—	—	—	—	—	—
Quaas et al. [14]	2021	Germany	—	Retrospective comparative study	6	458	F	148	—	44	95	29	40	40	20
							M	310	—	142	129	63	74	97	41
Sui et al. [85]	2021	China	Hospital	Retrospective comparative study	8	1496	F	435	59.5 ± 13.0/54.8 ± 12.02	88	347	—	—	—	—
							M	1061	61.9 ± 10.4/61.3 ± 11.6	364	697	—	—	—	—
Nam et al. [86]	2021	South Korea	Hospital	Retrospective comparative study	8	5961	F	1992	—	260	1730	1452	163	148	222
							M	3969	—	622	3340	2828	357	300	468
Kohlruss et al. [15]	2021	Germany	Hospital	Retrospective comparative study	8	717	F	188	—	75	99	—	—	—	—
							M	529	—	298	208	—	—	—	—
Ma et al. [87]	2021	China	Hospital	Retrospective comparative study	7	1404	F	477	—	—	—	—	—	—	—
							M	927	—	—	—	—	—	—	—
Dijksterhuis et al. [22]	2021	Netherlands	Community	Observational study	7	1836	F	719	—	—	—	—	—	—	—
							M	1117	—	—	—	—	—	—	—
Salari et al. [88]	2021	Iran	Hospital	Retrospective comparative study	6	186	F	51	—	—	—	—	—	—	—
							M	135	—	—	—	—	—	—	—
Choi et al. [16]	2022	South Korea	Hospital	Retrospective comparative study	7	2983	F	978	59.36 ± 13.47	19	959	—	—	—	—
							M	2005	61.66 ± 11.63	58	1947	—	—	—	—
Kiumarsi et al. [89]	2022	Iran	Hospital	Retrospective comparative study	6	314	F	79	—	—	—	—	—	—	—
							M	235	—	—	—	—	—	—	—

**Table 2:** Subgroup meta-analysis of clinicopathological characteristics between the female group and male group.

Group	Included studies	Included patients	*I* ^2^ (%)	Effect model	OR/WMD	95% CI	*P*
Female	61	700051	99	Random	0.27	[0.26, 0.29]	<0.00001
Age	7	11671	45	Fixed	−2.57	[−3.06, −2.09]	<0.00001
Lymph node metastasis							
N0	11	15891	36	Fixed	0.97	[0.91, 1.04]	0.44
N+	11	15891	35	Fixed	1.03	[0.96, 1.10]	0.48
pTNM stage							
I	12	44617	60	Random	1.01	[0.91, 1.13]	0.83
II	12	44617	65	Random	1.05	[0.93, 1.19]	0.43
III	11	44374	74	Random	0.97	[0.86, 1.10]	0.61
IV	9	39055	75	Random	0.89	[0.74, 1.06]	0.2
Tumor size	5	10507	77	Random	0.11	[−0.1, 0.33]	0.3
Tumor location							
Proximal	25	326452	97	Random	0.63	[0.53, 0.75]	<0.00001
Distal	25	326452	96	Random	1.41	[1.24, 1.60]	<0.00001
Total	5	21237	52	Random	1.34	[0.91, 1.96]	0.13
Overlapping	5	261081	98	Random	1.64	[1.02, 2.63]	0.04
Unknown/other	10	289212	98	Random	1.32	[1.07, 1.64]	0.01
Tumor stage							
Local	3	80919	94	Random	0.92	[0.69, 1.23]	0.59
Regional	3	80919	16	Fixed	1.05	[1.02, 1.08]	0.0009
Disseminated	3	80919	95	Random	0.95	[0.72, 1.25]	0.72
Missing	3	80919	44	Fixed	1.17	[1.12, 1.22]	<0.00001
Cardia	19	512322	99	Random	0.52	[0.44, 0.61]	<0.00001
Non-cardia	19	512322	99	Random	1.46	[1.26, 1.70]	<0.00001
Histologic grading							
Differentiation	5	13548	67	Random	0.44	[0.37, 0.51]	<0.00001
Undifferentiation	5	13548	67	Random	2.3	[1.98, 2.68]	<0.00001
Lauren type							
Intestinal	24	380595	98	Random	0.59	[0.49, 0.71]	<0.00001
Diffuse	23	367945	90	Random	1.87	[1.70, 2.06]	<0.00001
Other	16	333349	96	Random	1.18	[1.01, 1.37]	0.03
Histological differentiation							
Signet-ring cell	14	279154	84	Random	1.76	[1.55, 1.99]	<0.00001
Mucinous	11	266813	71	Random	1.06	[0.84, 1.32]	0.64
Borrmann							
I	6	11302	89	Random	1.03	[0.55, 1.94]	0.93
II	6	11302	54	Random	0.8	[0.68, 0.95]	0.009
III	6	11302	81	Random	0.86	[0.70, 1.06]	0.16
IV	6	11302	94	Random	1.41	[0.79, 2.50]	0.25
Complication	3	2912	0	Fixed	0.78	[0.66, 0.93]	0.005
Lymphovascular invasion	5	10725	78	Random	0.99	[0.79, 1.25]	0.96
Lymph node metastasis	3	5192	58	Random	0.96	[0.79, 1.17]	0.68
Hepatic metastasis	3	5192	0	Fixed	0.56	[0.47, 0.67]	<0.00001
Perineural metastasis	5	11137	90	Random	1.38	[0.90, 2.11]	0.14
Family history	4	7259	73	Random	1.30	[0.87, 1.93]	0.2
HP infection	3	5430	21	Fixed	1.16	[1.03, 1.31]	0.02

**Table 3:** Subgroup meta-analysis of survival outcomes between the female group and male group.

Group	Included studies	Included patients	*I* ^2^ (%)	Effect model	HR	95% CI	*P*
OS							
3-year OS	23	146597	53	Random	0.9	[0.86, 0.95]	0.0003
5-year OS	27	188166	66	Random	0.86	[0.82, 0.91]	<0.00001
OS of White patients							
3-year OS	7	69767	6	Fixed	0.88	[0.85, 0.91]	<0.00001
5-year OS	7	69767	0	Fixed	0.83	[0.80, 0.87]	<0.00001
OS of Asian patients							
3-year OS	14	56933	65	Random	0.95	[0.88, 1.03]	0.26
5-year OS	16	61814	77	Random	0.92	[0.84, 1.01]	0.08
OS of young patients							
1-year OS	6	2757	0	Fixed	1.13	[0.94, 1.35]	0.2
3-year OS	5	1974	57	Random	1.25	[0.91, 1.70]	0.17
5-year OS	6	2757	43	Fixed	1.39	[1.18, 1.65]	0.0001
OS of old patients							
1-year OS	7	57983	0	Fixed	0.93	[0.90, 0.97]	0.0005
3-year OS	6	24428	17	Fixed	0.91	[0.86, 0.97]	0.002
5-year OS	8	62214	78	Random	0.85	[0.76, 0.95]	0.005

## Data Availability

The data used to support the findings of this study are available from the corresponding author upon request.

## Supplementary Materials

Supplementary Materials Figure S1: meta-analysis of female ratio of total patients. Figure S2: meta-analysis of age between female and male groups. Figure S3: the proportion of clinicopathologic feature between female and male groups—(A) meta-analysis of proximal cancer; (B) meta-analysis of distal cancer; (C) meta-analysis of cardia cancer; (D) meta-analysis of non-cardia cancer; (E) meta-analysis of differentiation; (F) meta-analysis of undifferentiation; (G) meta-analysis of intestinal type; (H) meta-analysis of diffuse type; (I) meta-analysis of signet-ring cell carcinoma. Figure S4: meta-analysis of the proportion of HP infection history between female and male groups. Figure S5: meta-analysis of the proportion of postoperative complications between female and male groups. Figure S6: the 3-year and 5-year overall survival for gastric cancer between female and male groups among Asian gastric cancer patients—(A) the 3-year overall survival of Asian patients; (B) the 5-year overall survival of Asian patients. Figure S7: meta-analysis of the proportion of hepatic metastasis between female and male groups. Table S1: clinicopathological characteristics of the included studies.
