# Supplementary material for: Sex Disparity in Patients with Gastric Cancer: A Systematic Review and Meta-Analysis
Source: J Oncol. 2022 Nov 2;2022:1269435. doi: 10.1155/2022/1269435 (PMC9646304; doi:10.1155/2022/1269435)
Supplement: Supplementary Materials — Figure S1: meta-analysis of female ratio of total patients. Figure S2: meta-analysis of age between female and male groups. Figure S3: the proportion of clinicopathologic feature between female and male groups—(A) meta-analysis of proximal cancer; (B) meta-analysis of distal cancer; (C) meta-analysis of cardia cancer; (D) meta-analysis of non-cardia cancer; (E) meta-analysis of differentiation; (F) meta-analysis of undifferentiation; (G) meta-analysis of intestinal type; (H) meta-analysis of diffuse type; (I) meta-analysis of signet-ring cell carcinoma. Figure S4: meta-analysis of the proportion of HP infection history between female and male groups. Figure S5: meta-analysis of the proportion of postoperative complications between female and male groups. Figure S6: the 3-year and 5-year overall survival for gastric cancer between female and male groups among Asian gastric cancer patients—(A) the 3-year overall survival of Asian patients; (B) the 5-year overall survival of Asian patients. Figure S7: meta-analysis of the proportion of hepatic metastasis between female and male groups. Table S1: clinicopathological characteristics of the included studies. [file 1269435.f1.doc]

Supplements


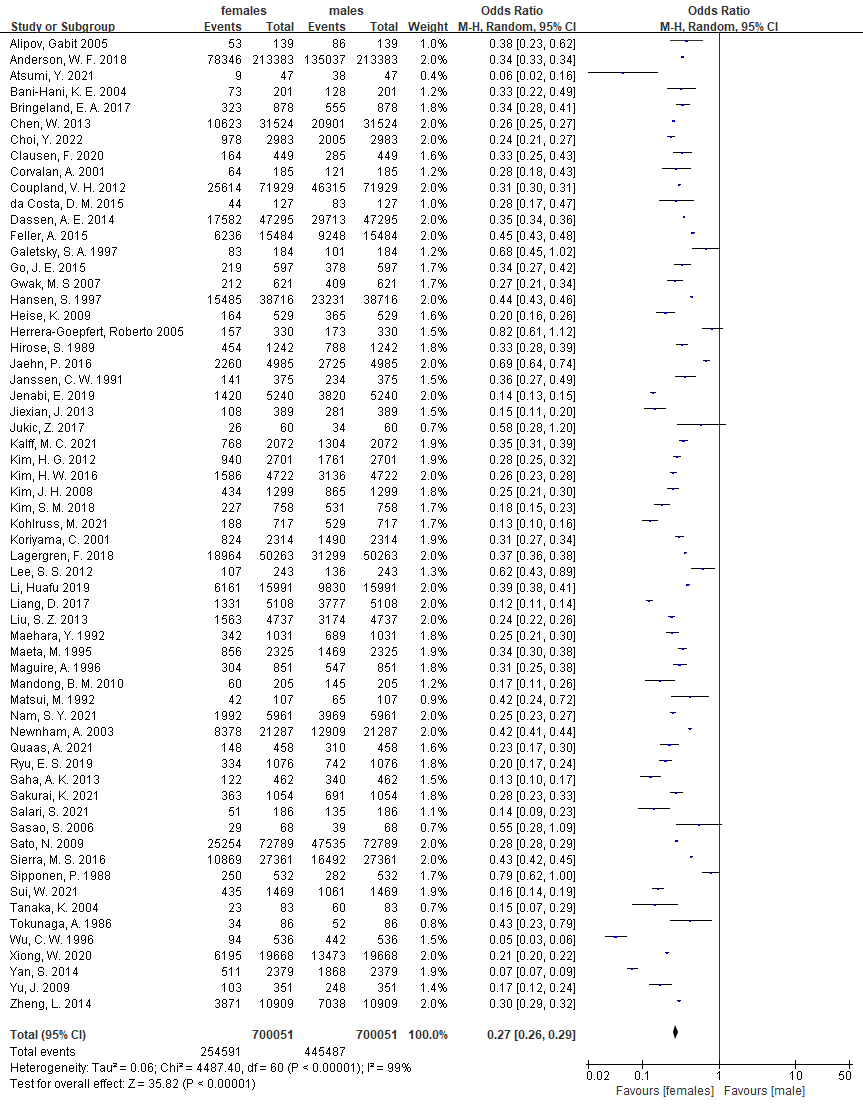


Figure S1 Meta-analysis of female ratio of total patients.


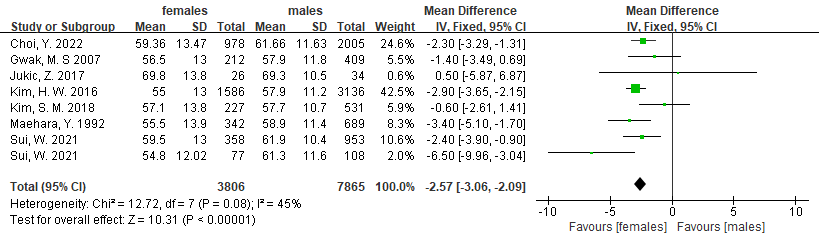


Figure S2 Meta-analysis of age between female and male group.

A

B

CD

E

F

G

H

I

Figure S3 The proportion of clinicopathologic feature between female and male group. A, Meta-analysis of proximal cancer; B, meta-analysis of distal cancer; C, meta-analysis of cardia cancer; D, meta-analysis of non-cardia cancer; E, meta-analysis of differentiation; F, meta-analysis of undifferentiation; G, meta-analysis of intestinal type; H, meta-analysis of diffuse type; I, meta-analysis of signet ring cell carcinoma.

Figure S4 Meta-analysis of the proportion of Hp infection history between female and male group.

Figure S5 Meta-analysis of the proportion of postoperative complications between female and male group.

A


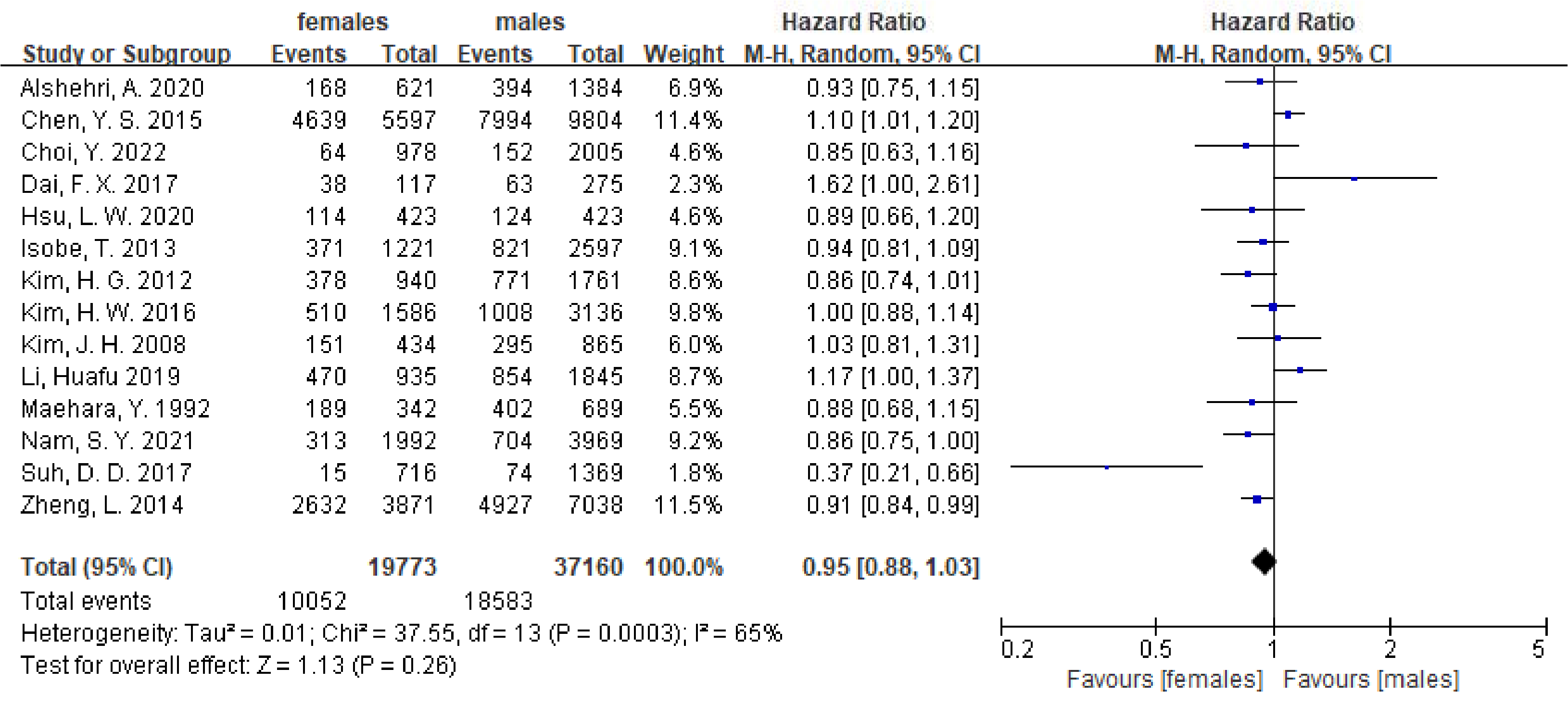


B


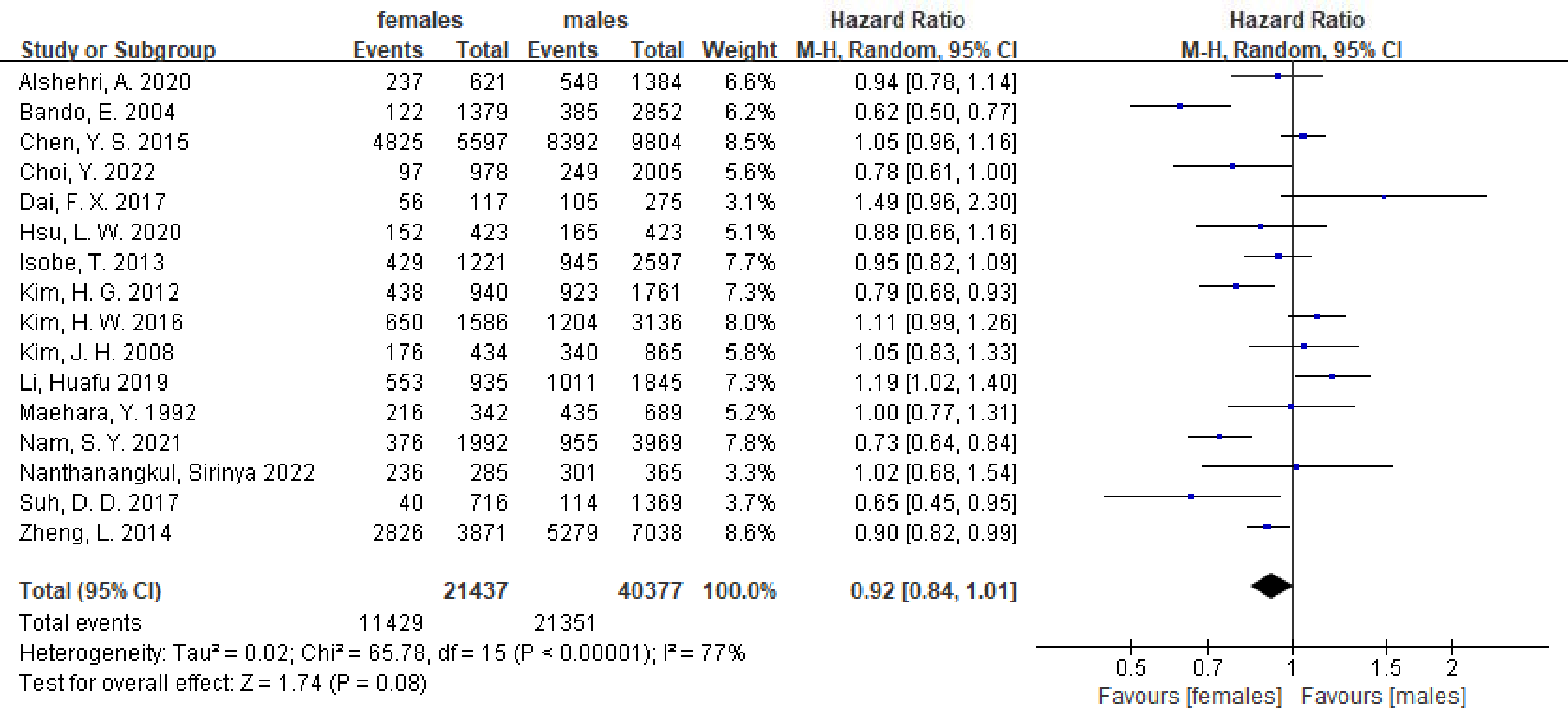


Figure S6 The 3-year and 5-year overall survival for gastric cancer between female and male group among Asian gastric cancer patients. A, The 3-year overall survival of Asian patients; B, the 5-year overall survival of Asian patients.

Figure S7 Meta-analysis of the proportion of hepatic metastasis between female and male group

Table S1 Clinicopathological characteristics of the included studies

| **Authors** | **Year** | **No.** | **Group** | | **Proximal** | | **Distal** | **Cardia** | **Non-cardia** | **Differentiation** | **undifferentiated** | **Intestinal** | **Diffuse** | **Signet-ring cell** | | **Tumor location** | | **TNM stage** | | | | **Complication** | **Hepatic metastasis** | **Hp infection** |
| --- | --- | --- | --- | --- | --- | --- | --- | --- | --- | --- | --- | --- | --- | --- | --- | --- | --- | --- | --- | --- | --- | --- | --- | --- |
|  |  |  |  |  |  |  |  |  |  |  |  |  |  |  |  | Proximal | Distal | I | II | III | IV |  |  |  |
| **Tokunaga, A.[50]** | 1986 | 86 | F | 34 | - | - | | - | - | - | - | 8 | 26 | | - | - | - | - | - | - | - | - | - | - |
|  |  |  | M | 52 | - | - | | - | - | - | - | 26 | 26 | | - | - | - | - | - | - | - | - | - | - |
| **Sipponen, P.[51]** | 1988 | 532 | F | 250 | 154 | 96 | | - | - | - | - | 111 | 139 | | - | 154 | 96 | - | - | - | - | - | - | - |
|  |  |  | M | 282 | 154 | 128 | | - | - | - | - | 162 | 120 | | - | 154 | 128 | - | - | - | - | - | - | - |
| **Hirose, S.[52]** | 1989 | 1242 | F | 454 | - | - | | - | - | - | - | - | 67 | | - | - | - | - | - | - | - | - | - | - |
|  |  |  | M | 788 | - | - | | - | - | - | - | - | 39 | | - | - | - | - | - | - | - | - | - | - |
| **Janssen, C. W.[53]** | 1991 | 375 | F | 141 | - | - | | - | - | - | - | 66 | 51 | | - | - | - | - | - | - | - | - | - | - |
|  |  |  | M | 234 | - | - | | - | - | - | - | 151 | 46 | | - | - | - | - | - | - | - | - | - | - |
| **Maehara, Y.[23]** | 1992 | 1031 | F | 342 | 86 | 256 | | - | - | 110 | 232 | - | - | | - | 86 | 256 | - | - | - | - | - | 18 | - |
|  |  |  | M | 689 | 207 | 482 | | - | - | 347 | 342 | - | - | | - | 207 | 482 | - | - | - | - | - | 56 | - |
| **Maeta, M.[49]** | 1995 | 2325 | F | 856 | - | - | | - | - | - | - | - | - | | - | - | - | - | - | - | - | - | 17 | - |
|  |  |  | M | 1469 | - | - | | - | - | - | - | - | - | | - | - | - | - | - | - | - | - | 64 | - |
| **Wu, C. W.[56]** | 1996 | 536 | F | 94 | - | - | | - | - | - | - | 27 | 63 | | - | - | - | - | - | - | - | - | - | - |
|  |  |  | M | 442 | - | - | | - | - | - | - | 241 | 168 | | - | - | - | - | - | - | - | - | - | - |
| **Maguire, A.[9]** | 1996 | 851 | F | 304 | - | - | | 13 | 291 | - | - | 118 | 31 | | - | - | - | - | - | - | - | - | - | - |
|  |  |  | M | 547 | - | - | | 36 | 511 | - | - | 243 | 62 | | - | - | - | - | - | - | - | - | - | - |
| **Galetsky, S. A.[57]** | 1997 | 184 | F | 83 | 11 | 69 | | 11 | 69 | - | - | - | - | | 4 | 11 | 69 | - | - | - | - | - | - | - |
|  |  |  | M | 101 | 37 | 61 | | 37 | 61 | - | - | - | - | | 3 | 37 | 61 | - | - | - | - | - | - | - |
| **Hansen, S.[69]** | 1997 | 38716 | F | 15485 | 1338 | 6020 | | - | - | - | - | - | - | | - | 1338 | 6020 | - | - | - | - | - | - | - |
|  |  |  | M | 23231 | 3048 | 8241 | | - | - | - | - | - | - | | - | 3048 | 8241 | - | - | - | - | - | - | - |
| **Koriyama, C.[58]** | 2001 | 2314 | F | 824 | 74 | 750 | | 74 | 750 | - | - | - | - | | - | 74 | 750 | - | - | - | - | - | - | - |
|  |  |  | M | 1490 | 204 | 1286 | | 204 | 1286 | - | - | - | - | | - | 204 | 1286 | - | - | - | - | - | - | - |
| **Corvalan, A.[59]** | 2001 | 185 | F | 64 | 22 | 42 | | 22 | 42 | - | - | 38 | 26 | | 6 | 22 | 42 | - | - | - | - | - | - | - |
|  |  |  | M | 121 | 45 | 73 | | 45 | 73 | - | - | 76 | 45 | | 3 | 45 | 73 | - | - | - | - | - | - | - |
| **Newnham, A.[70]** | 2003 | 21287 | F | 8378 | - | - | | 723 | 2059 | - | - | - | - | | - | - | - | - | - | - | - | - | - | - |
|  |  |  | M | 12909 | - | - | | 2025 | 3216 | - | - | - | - | | - | - | - | - | - | - | - | - | - | - |
| **Bani-Hani, K. E.[71]** | 2004 | 201 | F | 73 | - | - | | - | - | - | - | 37 | 20 | | - | - | - | - | - | - | - | - | - | - |
|  |  |  | M | 128 | - | - | | - | - | - | - | 90 | 9 | | - | - | - | - | - | - | - | - | - | - |
| **Tanaka, K.[67]** | 2004 | 83 | F | 23 | 3 | 20 | | - | - | - | - | - | - | | - | 3 | 20 | - | - | - | - | - | - | - |
|  |  |  | M | 60 | 11 | 49 | | - | - | - | - | - | - | | - | 11 | 49 | - | - | - | - | - | - | - |
| **Alipov, Gabit[72]** | 2005 | 139 | F | 53 | - | - | | - | - | - | - | 18 | 35 | | 12 | - | - | - | - | - | - | - | - | - |
|  |  |  | M | 86 | - | - | | - | - | - | - | 30 | 56 | | 15 | - | - | - | - | - | - | - | - | - |
| **Herrera-Goepfert, Roberto[73]** | 2005 | 330 | F | 157 | 13 | 143 | | - | - | - | - | 54 | 103 | | 39 | 13 | 143 | - | - | - | - | - | - | - |
|  |  |  | M | 173 | 31 | 141 | | - | - | - | - | 87 | 86 | | 32 | 31 | 141 | - | - | - | - | - | - | - |
| **Sasao, S.[60]** | 2006 | 134 | F | 53 | - | - | | - | - | - | - | 5 | 24 | | - | - | - | - | - | - | - | - | - | 26 |
|  |  |  | M | 81 | - | - | | - | - | - | - | 11 | 28 | | - | - | - | - | - | - | - | - | - | 34 |
| **Gwak, M. S.[61]** | 2007 | 621 | F | 212 | - | - | | - | - | - | - | - | - | | - | - | - | - | - | - | - | - | - | - |
|  |  |  | M | 409 | - | - | | - | - | - | - | - | - | | - | - | - | - | - | - | - | - | - | - |
| **Kim, J. H.[24]** | 2008 | 1299 | F | 434 | 39 | 384 | | - | - | - | - | - | - | | - | 39 | 384 | 197 | 61 | 123 | 53 | - | - | - |
|  |  |  | M | 865 | 100 | 754 | | - | - | - | - | - | - | | - | 100 | 754 | 321 | 104 | 230 | 210 | - | - | - |
| **Heise, K.[11]** | 2009 | 529 | F | 164 | 35 | 57 | | - | - | - | - | - | - | | 34 | 35 | 57 | 1 | 11 | 15 | 83 | - | - | - |
|  |  |  | M | 365 | 123 | 122 | | - | - | - | - | - | - | | 66 | 123 | 122 | 8 | 9 | 38 | 214 | - | - | - |
| **Yu, J.[62]** | 2009 | 351 | F | 103 | - | - | | - | - | - | - | - | - | | 27 | - | - | - | - | - | - | - | - | - |
|  |  |  | M | 248 | - | - | | - | - | - | - | - | - | | 32 | - | - | - | - | - | - | - | - | - |
| **Sato, N.[63]** | 2009 | 72789 | F | 25254 | - | - | | - | - | - | - | 6864 | 10401 | | - | - | - | - | - | - | - | - | - | - |
|  |  |  | M | 47535 | - | - | | - | - | - | - | 19493 | 14362 | | - | - | - | - | - | - | - | - | - | - |
| **Mandong, B. M.[74]** | 2010 | 205 | F | 60 | 25 | 35 | | 25 | 35 | - | - | - | - | | - | 25 | 35 | - | - | - | - | - | - | - |
|  |  |  | M | 145 | 45 | 100 | | 45 | 100 | - | - | - | - | | - | 45 | 100 | - | - | - | - | - | - | - |
| **Kim, H. G.[12]** | 2012 | 2701 | F | 940 | 72 | 857 | | - | - | - | - | - | - | | 102 | 72 | 857 | 444 | 194 | 255 | 47 | - | - | - |
|  |  |  | M | 1761 | 181 | 1565 | | - | - | - | - | - | - | | 145 | 181 | 1565 | 797 | 367 | 485 | 112 | - | - | - |
| **Lee, S. S.[30]** | 2012 | 243 | F | 107 | - | - | | - | - | - | - | - | - | | - | - | - | 80 | 27 | - | - | 11 | - | - |
|  |  |  | M | 136 | - | - | | - | - | - | - | - | - | | - | - | - | 113 | 23 | - | - | 19 | - | - |
| **Coupland, V. H.[75]** | 2012 | 71929 | F | 25614 | - | - | | 4621 | 5809 | - | - | - | - | | - | - | - | - | - | - | - | - | - | - |
|  |  |  | M | 46315 | - | - | | 14107 | 9531 | - | - | - | - | | - | - | - | - | - | - | - | - | - | - |
| **Saha, A. K.[76]** | 2013 | 462 | F | 122 | 24 | 84 | | - | - | - | - | - | - | | - | 24 | 84 | - | - | - | - | - | - | - |
|  |  |  | M | 340 | 52 | 242 | | - | - | - | - | - | - | | - | 52 | 242 | - | - | - | - | - | - | - |
| **Chen, W.[78]** | 2013 | 31524 | F | 10623 | - | - | | 640 | 9899 | - | - | 250 | 294 | | 1815 | - | - | - | - | - | - | - | - | - |
|  |  |  | M | 20901 | - | - | | 2527 | 18218 | - | - | 645 | 322 | | 1856 | - | - | - | - | - | - | - | - | - |
| **Liu, S. Z.[79]** | 2013 | 4737 | F | 1563 | - | - | | 999 | 233 | - | - | - | - | | - | - | - | - | - | - | - | - | - | - |
|  |  |  | M | 3174 | - | - | | 2014 | 501 | - | - | - | - | | - | - | - | - | - | - | - | - | - | - |
| **Yan, S.[80]** | 2014 | 2379 | F | 511 | - | - | | - | - | - | - | - | - | | 4 | - | - | - | - | - | - | - | - | 210 |
|  |  |  | M | 1868 | - | - | | - | - | - | - | - | - | | 4 | - | - | - | - | - | - | - | - | 757 |
| **Zheng, L.[42]** | 2014 | 10909 | F | 3871 | 418 | 1530 | | 366 | 1582 | - | - | - | - | | - | 418 | 1530 | 207 | 364 | 451 | 522 | - | - | - |
|  |  |  | M | 7038 | 1124 | 2705 | | 1005 | 2824 | - | - | - | - | | - | 1124 | 2705 | 394 | 721 | 902 | 982 | - | - | - |
| **Dassen, A. E.[81]** | 2014 | 47295 | F | 17582 | - | - | | 2709 | 14873 | - | - | - | - | | - | - | - | - | - | - | - | - | - | - |
|  |  |  | M | 29713 | - | - | | 8584 | 21129 | - | - | - | - | | - | - | - | - | - | - | - | - | - | - |
| **Feller, A.[82]** | 2015 | 15484 | F | 6236 | - | - | | 540 | 2287 | - | - | - | - | | - | - | - | - | - | - | - | - | - | - |
|  |  |  | M | 9248 | - | - | | 1668 | 2802 | - | - | - | - | | - | - | - | - | - | - | - | - | - | - |
| **da Costa, D. M.[64]** | 2015 | 127 | F | 44 | - | - | | 4 | 40 | - | - | - | - | | - | - | - | - | - | - | - | - | - | - |
|  |  |  | M | 83 | - | - | | 26 | 57 | - | - | - | - | | - | - | - | - | - | - | - | - | - | - |
| **Go, J. E.[31]** | 2015 | 597 | F | 219 | - | - | | - | - | - | - | - | - | | - | - | - | 182 | 26 | 11 | - | 19 | - | - |
|  |  |  | M | 378 | - | - | | - | - | - | - | - | - | | - | - | - | 340 | 36 | 2 | - | 45 | - | - |
| **Jaehn, P.[83]** | 2016 | 4985 | F | 2260 | - | - | | - | - | - | - | 915 | 778 | | - | - | - | - | - | - | - | - | - | - |
|  |  |  | M | 2725 | - | - | | - | - | - | - | 1417 | 768 | | - | - | - | - | - | - | - | - | - | - |
| **Sierra, M. S.[84]** | 2016 | 27361 | F | 10869 | - | - | | - | - | - | - | 7373 | 2553 | | - | - | - | - | - | - | - | - | - | - |
|  |  |  | M | 16492 | - | - | | - | - | - | - | 12236 | 2815 | | - | - | - | - | - | - | - | - | - | - |
| **Kim, H. W.[35]** | 2016 | 4722 | F | 1586 | - | - | | - | - | 662 | 924 | 245 | 295 | | 446 | - | - | 897 | 65 | 623 | - | - | - | - |
|  |  |  | M | 3136 | - | - | | - | - | 1867 | 1269 | 758 | 416 | | 490 | - | - | 1858 | 119 | 1159 | - | - | - | - |
| **Liang, D.[85]** | 2017 | 5108 | F | 1331 | 787 | 390 | | 713 | 464 | - | - | - | - | | - | 787 | 390 | - | - | - | - | - | - | - |
|  |  |  | M | 3777 | 2512 | 905 | | 2331 | 1086 | - | - | - | - | | - | 2512 | 905 | - | - | - | - | - | - | - |
| **Jukic, Z.[65]** | 2017 | 60 | F | 26 | - | - | | - | - | - | - | - | - | | - | - | - | - | - | - | - | - | - | - |
|  |  |  | M | 34 | - | - | | - | - | - | - | - | - | | - | - | - | - | - | - | - | - | - | - |
| **Bringeland, E. A.[86]** | 2017 | 878 | F | 323 | - | - | | 38 | 230 | - | - | 130 | 132 | | - | - | - | - | - | - | - | - | - | - |
|  |  |  | M | 555 | - | - | | 186 | 309 | - | - | 304 | 135 | | - | - | - | - | - | - | - | - | - | - |
| **Kim, S. M.[7]** | 2018 | 758 | F | 227 | 9 | 218 | | - | - | 44 | 183 | 49 | 178 | | - | 9 | 218 | 137 | 45 | 39 | 6 | - | - | - |
|  |  |  | M | 531 | 70 | 461 | | - | - | 203 | 328 | 221 | 310 | | - | 70 | 461 | 328 | 80 | 117 | 6 | - | - | - |
| **Anderson, W. F.[87]** | 2018 | 142783 | F | 63746 | 20553 | 31408 | | 16287 | 35674 | - | - | 3972 | 8399 | | 7179 | 20553 | 31408 | - | - | - | - | - | - | - |
|  |  |  | M | 79037 | 67920 | 36288 | | 62222 | 41986 | - | - | 5542 | 7639 | | 6425 | 67920 | 36288 | - | - | - | - | - | - | - |
| **Lagergren, F.[88]** | 2018 | 50263 | F | 18964 | - | - | | 1630 | 17334 | - | - | - | - | | - | - | - | - | - | - | - | - | - | - |
|  |  |  | M | 31299 | - | - | | 5288 | 26011 | - | - | - | - | | - | - | - | - | - | - | - | - | - | - |
| **Jenabi, E.[89]** | 2019 | 5240 | F | 1420 | - | - | | - | - | - | - | - | - | | 243 | - | - | - | - | - | - | - | - | - |
|  |  |  | M | 3820 | - | - | | - | - | - | - | - | - | | 536 | - | - | - | - | - | - | - | - | - |
| **Ryu, E. S.[47]** | 2019 | 1076 | F | 334 | - | - | | - | - | 102 | 232 | - | - | | - | - | - | - | - | - | - | - | - | - |
|  |  |  | M | 742 | - | - | | - | - | 443 | 299 | - | - | | - | - | - | - | - | - | - | - | - | - |
| **Li, Huafu[44]** | 2019 | 15991 | F | 6161 | 918 | 3857 | | 706 | 4069 | - | - | 400 | - | | 1028 | 918 | 3857 | 449 | 1597 | 3907 | 211 | - | - | - |
|  |  |  | M | 9830 | 2068 | 5259 | | 1741 | 5586 | - | - | 653 | - | | 1146 | 2068 | 5259 | 639 | 2930 | 5913 | 348 | - | - | - |
| **Clausen, F.[40]** | 2020 | 449 | F | 164 | 39 | 125 | | - | - | - | - | 64 | 79 | | - | 39 | 125 | 29 | 42 | 54 | 39 | - | - | - |
|  |  |  | M | 285 | 104 | 172 | | - | - | - | - | 175 | 57 | | - | 104 | 172 | 45 | 59 | 135 | 46 | - | - | - |
| **Xiong, W.[90]** | 2020 | 19668 | F | 6195 | 850 | 1515 | | 706 | 1831 | - | - | - | - | | - | 850 | 1515 | - | - | - | - | - | - | - |
|  |  |  | M | 13473 | 3516 | 2750 | | 3205 | 3552 | - | - | - | - | | - | 3516 | 2750 | - | - | - | - | - | - | - |
| **Kalff, M. C.[32]** | 2021 | 2072 | F | 768 | - | - | | - | - | - | - | 239 | 258 | | - | - | - | - | - | - | - | 252 | - | - |
|  |  |  | M | 1304 | - | - | | - | - | - | - | 515 | 311 | | - | - | - | - | - | - | - | 496 | - | - |
| **Quaas, A.[14]** | 2021 | 458 | F | 148 | 44 | 95 | | - | - | - | - | - | - | | - | 44 | 95 | 29 | 40 | 40 | 20 | - | - | - |
|  |  |  | M | 310 | 142 | 129 | | - | - | - | - | - | - | | - | 142 | 129 | 63 | 74 | 97 | 41 | - | - | - |
| **Sui, W.[48]** | 2021 | 1496 | F | 435 | 88 | 347 | | - | - | - | - | 272 | 111 | | 61 | 88 | 347 | - | - | - | - | - | - | - |
|  |  |  | M | 1061 | 364 | 697 | | - | - | - | - | 779 | 156 | | 65 | 364 | 697 | - | - | - | - | - | - | - |
| **Nam, S. Y.[41]** | 2021 | 5961 | F | 1992 | 260 | 1730 | | - | - | 769 | 1222 | - | - | | - | 260 | 1730 | 1452 | 163 | 148 | 222 | - | - | - |
|  |  |  | M | 3969 | 622 | 3340 | | - | - | 2239 | 1717 | - | - | | - | 622 | 3340 | 2828 | 357 | 300 | 468 | - | - | - |
| **Kohlruss, M.[15]** | 2021 | 717 | F | 188 | 75 | 99 | | - | - | - | - | 82 | - | | - | 75 | 99 | - | - | - | - | - | - | - |
|  |  |  | M | 529 | 298 | 208 | | - | - | - | - | 315 | - | | - | 298 | 208 | - | - | - | - | - | - | - |
| **Dijksterhuis, W. P. M.[22]** | 2021 | 1836 | F | 719 | - | - | | - | - | - | - | - | - | | - | - | - | - | - | - | - | - | 177 | - |
|  |  |  | M | 1117 | - | - | | - | - | - | - | - | - | | - | - | - | - | - | - | - | - | 406 | - |
| **Choi, Y.[16]** | 2022 | 2983 | F | 978 | 19 | 959 | | - | - | - | - | 447 | 447 | | - | 19 | 959 | - | - | - | - | - | - | 599 |
|  |  |  | M | 2005 | 58 | 1947 | | - | - | - | - | 1396 | 520 | | - | 58 | 1947 | - | - | - | - | - | - | 1117 |
